# The uniportal VATS in the treatment of stage II pleural empyema: a safe and effective approach for adults and elderly patients—a single-center experience and literature review

**DOI:** 10.1186/s13017-022-00438-8

**Published:** 2022-08-29

**Authors:** Claudio Luciani, Andrea Scacchi, Roberto Vaschetti, Giancarlo Di Marzo, Ilaria Fatica, Micaela Cappuccio, Germano Guerra, Graziano Ceccarelli, Pasquale Avella, Aldo Rocca

**Affiliations:** 1 General Surgery Unit, A. Cardarelli Hospital, Campobasso, CB Italy; 2 Department of Medicine and Health Science, University of Molise, Campobasso, Italy; 3 General and Robotic Surgery Department, San Giovanni Battista Hospital, Foligno, Perugia, Italy

**Keywords:** Thoracic surgery, Pleural empyema, Minimally invasive surgery, Frailty, Uniportal video-assisted thoracoscopy, Decortication

## Abstract

**Background:**

Pleural empyema (PE) is a frequent disease, associated with a high morbidity and mortality. Surgical approach is the standard of care for most patients with II-III stage PE. In the last years, the minimally invasive surgical revolution involved also thoracic surgery allowing the same outcomes in terms of safety and effectiveness combined to better pain management and early discharge. The aim of this study is to demonstrate through our experience on uniportal-video-assisted thoracoscopy (u-VATS) the effectiveness and safety of its approach in treatment of stage II PE. As secondary endpoint, we will evaluate the different pattern of indication of u-VATS in adult and elderly patients with literature review.

**Methods:**

We retrospectively reviewed our prospectively collected database of u-VATS procedures from November 2018 to February 2022, in our regional referral center for Thoracic Surgery of Regione Molise General Surgery Unit of “A. Cardarelli” Hospital, in Campobasso, Molise, Italy.

**Results:**

A total of 29 patients underwent u-VATS for II stage PE. Fifteen (51.72%) patients were younger than 70 years old, identified as “adults,” 14 (48.28%) patients were older than 70 years old, identified as “elderly.” No mortality was found. Mean operative time was 104.68 ± 39.01 min in the total population. The elderly group showed a longer operative time (115 ± 53.15 min) (*p* = *0.369*). Chest tube was removed earlier in adults than in elderly group *(5.56* ± 2.06 *vs.* 10.14 ± 5.58 *p* = *0.038).* The Length of Stay (LOS) was shorter in the adults group (6.44 ± 2.35 *vs.* 12.29 ± 6.96 *p* = *0.033*). Patients evaluated through Instrumental Activities of Daily Living (IADL) scale returned to normal activities of daily living after surgery.

**Conclusion:**

In addition, the u-VATS approach seems to be safe and effective ensuring a risk reduction of progression to stage III PE with a lower recurrence risk and septic complications also in elderly patients. Further comparative multicenter analysis are advocated to set the role of u-VATS approach in the treatment of PE in adults and elderly patients.

**Supplementary Information:**

The online version contains supplementary material available at 10.1186/s13017-022-00438-8.

## Background

Pleural empyema (PE) is a clinical condition defined as the presence of purulent fluid in the pleural cavity and characterized by high morbidity and an estimated mortality rate of 15% [1].

The surgical approach to PE is determined by its evolutive stage and it is the standard of care in the 36–65% of patients [2].

Empyema thoracis is classified into three stages, according to the European Association for Cardio-Thoracic Surgery (EACTS) guidelines [3]: the exudative phase (stage I) is characterized by a collection of flowing fluid into the pleural cavity in the absence of positive culture. The fibrinopurulent phase (stage II), a turbid and frankly infected fluid, with loculations and fibrinous septa formation. Lastly, the chronic organizing phase (stage III), with scar adhesions and a progressive constriction, process that leads to a “trapped lung.”

EACTS guidelines recommend parenteral antibiotics combined to image-guided pleural drain placement for the first stage of PE, instead, surgery should be considered the first-line approach in the management of the last stages when single lung ventilation is tolerated either in minimally invasive approach, either through open surgery [1, 3]. In cases where such surgical intervention or ventilation cannot be tolerable for the patient, intrapleural fibrinolysis may be an alternative approach [3].

The advent of minimally invasive surgery (MIS) approaches and technological improvements have led to significant amelioration in peri-operative patients’ outcomes also in complex surgical fields [4–8]. Following the general surgery experience in MIS approaches also thoracic surgery have demonstrated the safety and efficacy of surgical approach through minimally invasive video-assisted thoracoscopy (VATS).

Nowadays, multiportal VATS (m-VATS), and its evolution uniportal-VATS (u-VATS), gained a key role in diagnosis and treatment of pulmonary and non-pulmonary morbidities [9, 10].

Furthermore, also the u-VATS approach to pulmonary cancers and pleural disease seemed to be related to enough safe and effective allowing better postoperative outcomes when compared to standard thoracotomy [2, 11, 12].

Although an increasing number of centers have reported VATS approach to treat intermediate stages of PE a lack of published data still persists on the use of u-VATS.

Therefore, our aim is to demonstrate through our experience on u-VATS the effectiveness and safety of its approach in treatment of stage II PE. As secondary endpoint we will evaluate the different pattern of indication of this both in adult and elderly patients with an extensive literature review.

## Methods

**Fig. 1:**
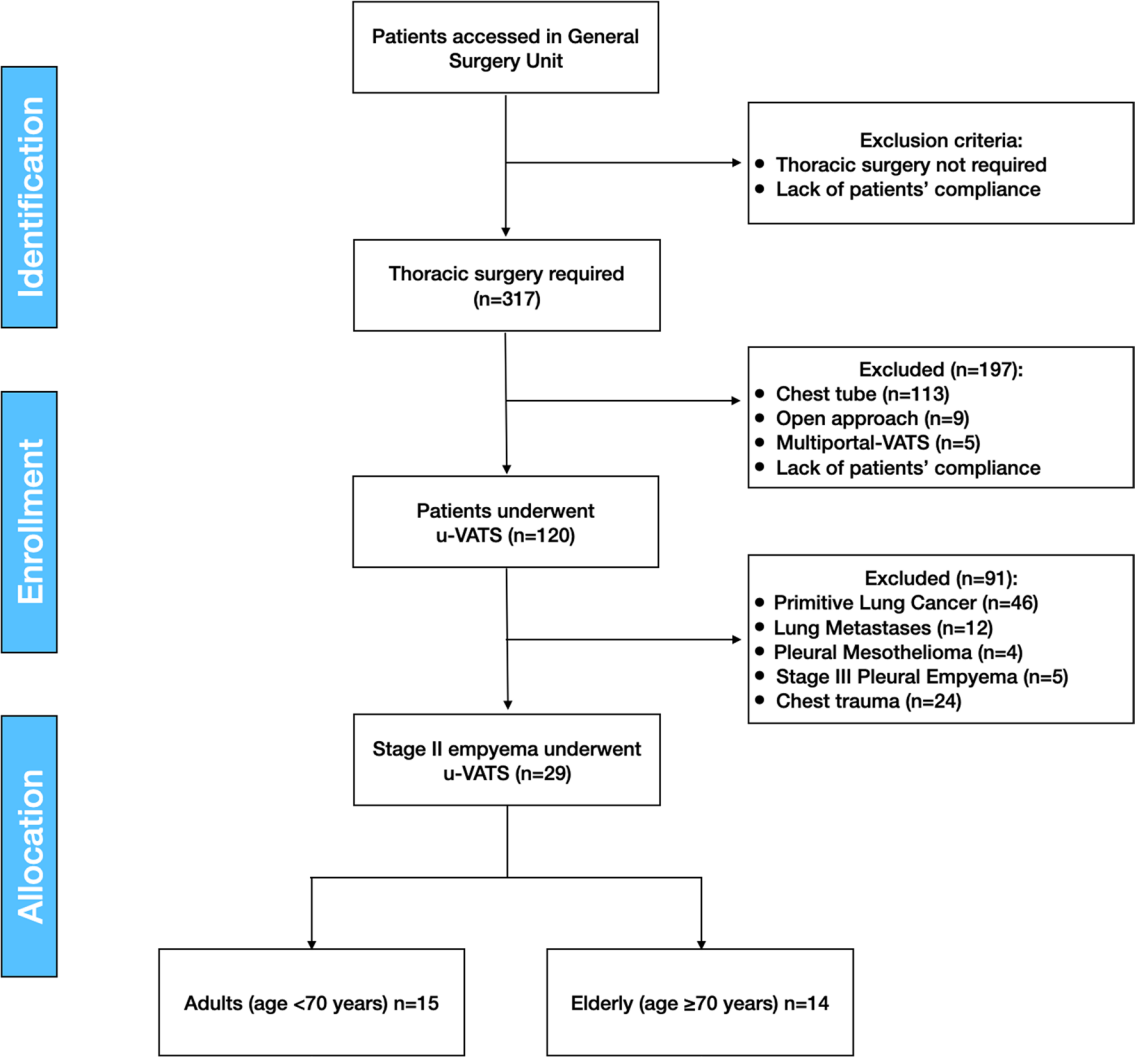
STROBEline flowchart. u-VATS, uniportal-video-assisted thoracoscopy; COPD, chronic obstructive pulmonary disease

We retrospectively reviewed our prospectively collected database of u-VATS procedures from the institution of a dedicated thoracic surgical team on November 2018 to February 2022, according to Strengthening the Reporting of Observational studies in Epidemiology (STROBE) [13], in our regional referral center for Thoracic Surgery of Regione Molise General Surgery Unit of “A. Cardarelli” Hospital, in Campobasso, Molise, Italy (Fig. 1).

**Fig. 2:**
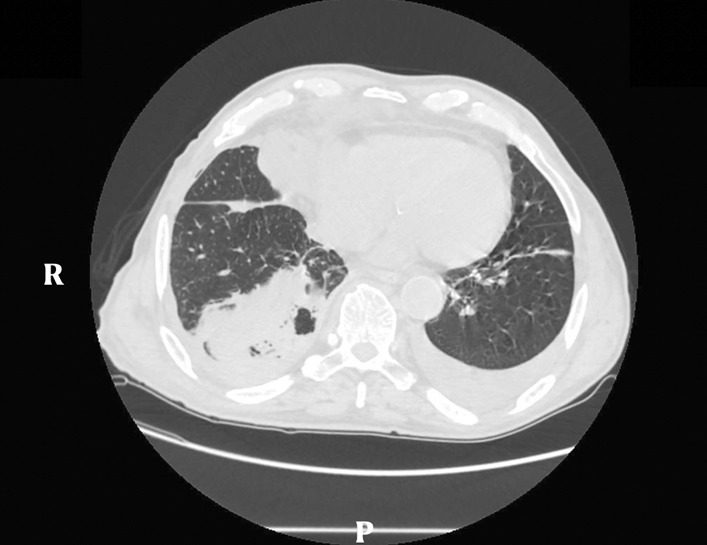
Computed tomography scan of the chest showing a right pleural effusion

Grade of eligibility was defined by the presence of a stage II PE fitting for surgery, according to the American Association of Thoracic Surgery (AATS) classification, evaluated through biochemical investigations, such as the alterations of flogosis indices, and instrumental investigations, like ultrasound (US), chest-XR and computed tomography (CT) scan [1] (Fig. 2).

Surgical exclusion criteria were: lack of patient compliance, the presence of lung cancer, chest trauma, non-pulmonary surgery and stage III PE.

Anesthesiological exclusion criteria were: lack of patient compliance, right ventricular dysfunction, hemodynamic instability, severe chronic obstructive pulmonary disease (COPD), severe pulmonary hypertension and other comorbidities which make it impossible to perform a single lung ventilation (Fig. 1).

Study exclusion criteria were: patients managed by general surgeons not involved in thoracic team before its institution, any data not prospectively collected.

A team composed of thoracic surgeons, anesthesiologist, infectious disease specialist and internist/pulmonologist discussed all cases, placing surgical indication, according to the EACTS guidelines [3]. American Society of Anesthesiologist (ASA) score was used to evaluate intraoperative risk [14].

Before surgery, all patients performed routine blood samples, electrocardiogram (ECG), and start a broad-spectrum antibiotic therapy including metronidazole with parental second or third cephalosporin generation (more frequent ceftriaxone) or parental aminopenicillin with ß-lactamase inhibitor (more frequent ampicillin/sulbactam) according to AATS guideline [1].

Due to the high incidence of the severe acute respiratory syndrome corona virus-2 (SARS-CoV-2) over the years of this study, all patients performed a rhino-pharyngeal molecular swab before the admission in the general surgery ward.

After surgery, all patients performed a chest-XR or CT scan to visualize lung re-expansion.

To achieve a rapid recovery of patient’s conditions, the enhanced recovery after surgery (ERAS) program was used [15].

Postoperative complications were assessed according to the Clavien–Dindo classification [16].

Treatment of postoperative anemia, a frequent complication during thoracic surgery, was performed according to our general surgery unit protocol (Additional file 1).

Postoperative pain was evaluated, every morning, through Visual Analogue Scale (VAS) [17].

Follow-up was planned at 1 week after discharge, using lung US, and 30 days, using chest-XR, after discharge. In either adult and elderly patients, Instrumental Activities of Daily Living (IADL) scale was administered in order to understand the return to normal activities of daily living [18].

All individuals included in this study signed an informed consent for the scientific anonymous use of clinical data. The study was conducted according to the guidelines of the Declaration of Helsinki and approved by the Institutional Review Board of the University of Molise (protocol number 10/21, approved date: May 12, 2021).

### Technical Notes

#### u-VATS Technique

All procedures were performed under general anesthesia and single lung ventilation, using a double-lumen endotracheal tube. The patient was put in lateral decubitus position with the arms flexed toward the head. To allow a better intercostal space extension, the operating table was flexed into a wedge position with the patient’s head and lower limbs slightly inclined.

**Fig. 3:**
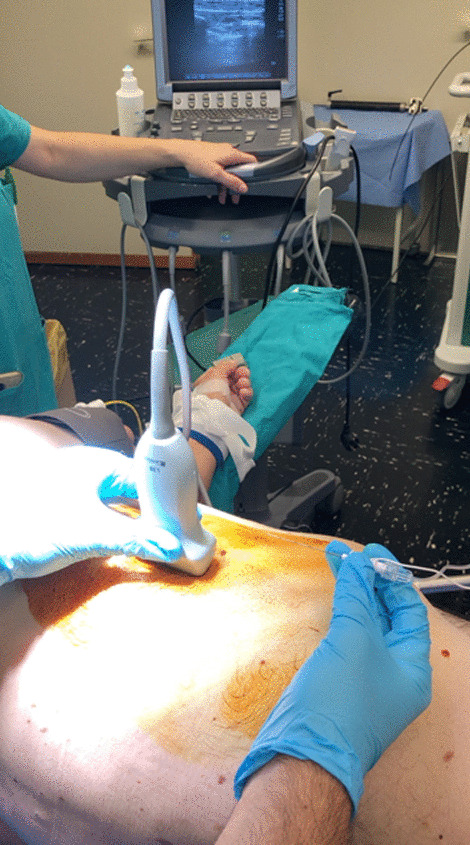
Ultrasonographic evaluation in serratus plane block (SAP block) during uniportal video-assisted thoracoscopy (u-VATS) surgery

After careful skin disinfection with iodopovidone 10%, an ultrasound-guided block of the serratus anterior plane (SAP block with ropivacaine 0.25%, 30 ml) was performed to achieve a better postoperative pain management (Fig. 3).

The operating surgeon and the assistant stand in front of the patient and the video-monitor.

**Fig. 4:**
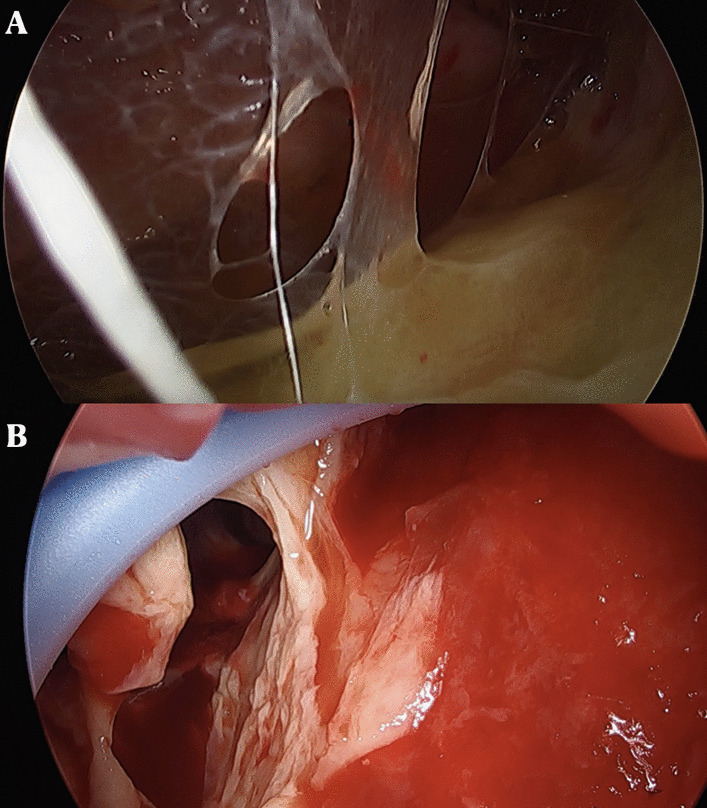
**A**, **B** Stage II of pleural empyema at uniportal video-assisted thoracoscopy (u-VATS) approach characterized by a exudative thickening and dense fibrin depositions in pleural space

At the level of the fifth intercostal space, in the correspondence of the mid axillary line, a single incision of 2–3 cm, preserving the muscular structure, was made. Subsequently, to ensure incision enlargement and protection of the structure, a wound protector was inserted. At this point, a 5 mm or 10 mm 30° thoracoscope and endoscopic instruments were introduced (Fig. 4).

**Fig. 5:**
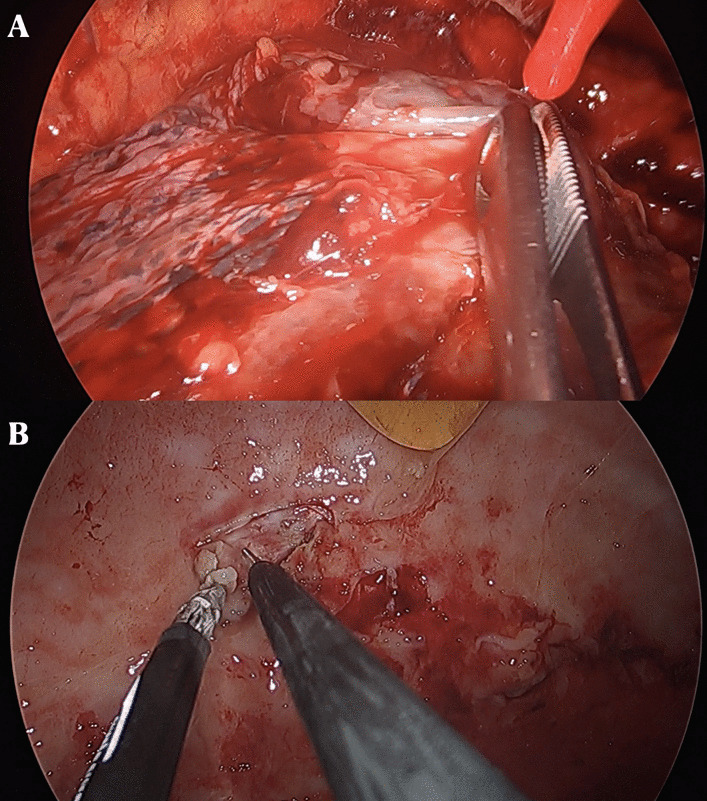
**A**, **B** Visceral and parietal decortication in patient affected by stage II pleural empyema

To achieve a complete lung re-expansion, the operation continued with debridement and removal of all adhesions, septa, and inflammatory effusion from both the visceral and the parietal pleura (Fig. 5).

In case of difficulty in operating management through thoracoscopy, an anterolateral thoracotomy was performed.

Multiple washings with warm physiological solution (NaCl 0.9% solution) were performed to eliminate the residual effusion and organized pus from the visceral pleura. The operation proceeded with decortication with an electrocautery device.

Parenchymal re-expansion was evaluated with lung inflation, and at the same time an accurate aerostasis’ control was carried out.

At the end of surgery, one or two chest tube drainages (28–32 French) were placed.

The timing of chest tube removal was inspired by post-surgical factors (no air leak, drained fluid of clear appearance, whose quantity in 24 h was less than 450 ml) [15], clinical aspects (quantitative decrease of inflammatory indices, fever) and radiological evidence of complete lung re-expansion.

Postoperatively, patients continued with the antibiotic regimen instituted preoperatively and analgesics, such as paracetamol (1000 mg/100 ml) or ketorolac (30 mg/ml), as needed for pain control. Gradually then, patients were referred to respiratory physiotherapy.

### Statistical analysis

Statistical analysis was designed to better define the safety and effectiveness of u-VATS in both adults (age < 70 years) and elderly (age ≥ 70 years).

A two-tailed *p value* < 0.05 was accepted as statistically significant.

First, we applied the Shapiro–Wilk test, to test the normal distribution of quantitative elements. Later, independent samples, whose distribution was normal, were tested using the unpaired t-test. The Mann–Whitney test was used in case of non-normal distributions of values.

Quantitative data were expressed like mean ± standard deviation (SD). We used the random-effects model to calculate summary 95% confidence interval (C.I.).

Chi-square test (χ^2^) or Fisher’s exact test was used for qualitative data.

Data analysis was carried out with IBM Statistical Package for the Social Sciences (IBM SPSS®).

## Results

Twenty-nine patients, 21 (72.41%) male and 8 (27.59%) female, underwent u-VATS surgery from November 2018 to February 2022. Fifteen (51.72%) patients were younger than 70 years old defined as “adults,” and 14 (48.28%) were elderly than 70 years defined as “elderly.” Total population mean age was 67.13 ± 9.96 years (95% C.I. 63.34–70.91). In the adults group, the mean age was 59.84 ± 5.34 (95% C.I. 56.88–62.79); meanwhile, in the elderly group the mean age was 76.51 ± 5.25 (95% C.I. 73.47–79.54). A statistically significant difference was found between the average ages of the two groups (*p* < *0.001*).

Hypertension (68.96%) was the most frequent comorbidity, both in adults (66.67%) and in elderly (71.42%). According to the ASA score, 12 (41.38%) patients were ASA 2, 11 (37.93%) were ASA 3, and 6 (20.69%) were ASA 4 (*p* = *0.017*). ASA score according to age is reported in Table 1.

**Table 1:** Baseline characteristic of patients

Variables	N. (%) and/or Mean ± SD			
	Total population (n. = 29)	Adults (age < 70; n. = 15)	Elderly (age ≥ 70; n. = 14)	*p* value
Age (years)	67.13 ± 9.96	59.84 ± 5.34	76.51 ± 5.25	< *0.001*
Gender				*0.035*
Male	21 (72.41)	8 (53.33)	13 (92.86)	
Female	8 (27.59)	7 (46.67)	1 (7.14)	
ASA				*0.017*
II	12 (41.38)	8 (53.33)	4 (28.57)	
III	11 (37.93)	7 (46.67)	4 (28.57)	
IV	6 (20.69)	0 (0)	6 (42.86)	
Smoker	13 (44.83)	7 (46.67)	6 (42.86)	*0.873*
Comorbidities				
Hypertension	20 (68.96)	10 (66.67)	10 (71.42)	
COPD	10 (34.48)	2 (13.33)	8 (57.14)	*0.021*
Heart disease	10 (34.48)	2 (13.33)	8 (57.14)	*0.021*
Diabetes	4 (13.79)	2 (13.33)	2 (14.29)	
SARS-CoV-2 swab positivity	0 (0)	0 (0)	0 (0)	
Pleural empyema stage				
I	0 (0)	0 (0)	0 (0)	
II	29 (100)	15 (100)	14 (100)	
III	0 (0)	0 (0)	0 (0)	
Affected lung				
Left	20 (68.97)	12 (80.00)	8 (57.14)	
Right	9 (31.03)	3 (20.00)	6 (42.86)	

SD, standard deviation; ASA, American Society of Anaesthesiologists Physical Score; COPD, chronic obstructive pulmonary disease; SARS-CoV-2, severe acute respiratory syndrome coronavirus 2

All PE (100%) were stage II affecting left hemithorax in the 68.97%.

The main patient’s demographic and clinical characteristics are shown in Table 1.

Five (17.24%) patients had parapneumonic empyema related to an ongoing acute pulmonary infectious process, 3 (20.00%) of these patients were adults.

Fever, at the admission, was the main symptom in 44.83% of the series, as shown in Table 2.

**Table 2:** Pre-, intra- and postoperative course and characteristics

Variables	N. (%) and/or Mean ± SD			
	Total population (n. = 29)	Adults (age < 70; n. = 15)	Elderly (age ≥ 70; n. = 14)	*p* value
*Preoperative characteristic*				
Preoperative pulmonary infection	5 (17.24)	3 (20.00)	2 (14.29)	
Clinical presentation				
Fever	13 (44.83)	3 (20.00)	10 (71.42)	*0.005*
Cough	4 (13.79)	0 (0)	4 (28.57)	*0.042*
Dyspnea	6 (20.69)	2 (13.33)	4 (28.57)	*0.390*
Preoperative flogosis indices				
WBC count (× 10^3^/uL)	10.84 ± 4.33	11.54 ± 3.34	10.14 ± 5.31	*0.565*
PCR (mg/L)	142.26 ± 131.77	87.60 ± 128.70	210.60 ± 114.06	*0.190*
PCT (ng/mL)	2.16 ± 3.10	3.50 ± 4.66	1.49 ± 2.64	*0.533*
Preoperative treatment				
Antibiotic therapy	29 (100)	15 (100)	14 (100)	
Corticosteroids	5 (17.24)	5 (33.33)	0 (0)	
Preoperative chest tube	9 (31.03)	7 (46.67)	2 (14.29)	*0.109*
Preoperative fibrinolytic therapy	0 (0)	0 (0)	0 (0)	
Preoperative lung re-expansion	0 (0)	0 (0)	0 (0)	
*Intraoperative course*				
Conversion to thoracotomy	1 (3.45)	1 (6.67)	0 (0)	
Number of chest tube				
1	25 (86.20)	13 (86.67)	12 (85.71)	
2	4 (13.80)	2 (13.33)	2 (14.29)	
Estimated blood loss (ml)	118 ± 73.5	110 ± 63	126 ± 84	
Re-expansion of the lung	29 (100)	15 (100)	14 (100)	
Operating time	104.68 ± 39.01	96.67 ± 23.84	115 ± 53.15	*0.369*
*Postoperative course*				
Clavien–Dindo classification				
I	18 (62.07)	6 (40.00)	12 (85.71)	
II	0 (0)	0 (0)	0 (0)	
III	0 (0)	0 (0)	0 (0)	
IV	0 (0)	0 (0)	0 (0)	
ICU admission	0 (0)	0 (0)	0 (0)	
Postoperative antibiotic therapy	29 (100)	15 (100)	14 (100)	
Postoperative Hb value (g/dL)	8.80 ± 0.96	9.40 ± 0.87	8.20 ± 1.06	
LOS (days)	9.00 ± 5.59	6.44 ± 2.35	12.29 ± 6.96	*0.033*
Mean pain duration (days)	2.80 ± 0.83	2.50 ± 0.70	3.00 ± 1.00	*0.591*
Painkillers needed	17 (58.62)	7 (46.67)	10 (71.43)	*0.167*
Chest tube removal (days)	7.56 ± 4.50	5.56 ± 2.06	10.14 ± 5.58	*0.038*

SD, standard deviation; WBC, white blood cell; PCR, protein-C reactive; PCT, procalcitonin; ICU, intensive care unit; Hb, hemoglobin; LOS, length of stay

The difference in the presence of fever between adults and elderly has been found as statistically significant (*p* = *0.005*). The presence of a cough as a clinical presentation was observed in 4 (100%) patients, all included in the elderly group (*p* = *0.042*).

All patients (100%) were treated with broad-spectrum antibiotic therapy; 5 (33.33%) adults patients were also administered a corticosteroid therapy.

Mean operative time was 104.68 ± 39.01 (95% C.I. 89.84–119.52) min in the total population. The elderly group showed a longer operative time (115 ± 53.15 min, 95% C.I. 84.31–145.69) without any statistical significance (*p* = *0.369*) (Table 2).

Only in 1 (3.45%) adult case conversion was necessary.

Table 2 illustrates the main preoperative, intraoperative, and postoperative characteristics. All patients (100%) achieved optimal lung re-expansion, and no air leaks were reported in any patient, at radiographic control.

No intraoperative or postoperative hemotransfusions were required.

No intraoperative mortality was reported.

No intensive care unit (ICU) admission was necessary (Table 2).

Postoperative complications according to Clavien–Dindo classification are depicted in Table 2.

Minor complications occurred in 18 (62.07%) patients, atelectasis was the most reported minor complication, 7 (46.67%) cases in the adults group, and 8 (57.14%) in the elderly group (*p* = *0.573*) (Table 3).

**Table 3:** Minor complications presentation

Variables	N. (%) and/or Mean ± SD			
	Total population (n. = 29)	Adults (age < 70; n. = 15)	Elderly (age ≥ 70; n. = 14)	*p* value
Atelectasis	14 (48.27)	7 (46.67)	8 (57.14)	*0.573*
Anemia	13 (44.83)	3 (20.00)	10 (71.42)	*0.005*
Air-leakage	0 (0)	0 (0)	0 (0)	
Wound infection	0 (0)	0 (0)	0 (0)	
Postoperative paresthesia	0 (0)	0 (0)	0 (0)	

SD, standard deviation

No surgical wound infections or local paresthesia occurred, as shown in Table 3.

Mean pain duration after surgery was 2.80 ± 0.83 days (95% C.I. 2.49–3.11). Seventeen (58.62%) patients, 7 (46.67%) in the adults group, and 10 (71.43%) in the elderly group required additional postoperative antalgic coverage.

Chest tube was removed after 7.56 ± 4.50 days (95% C.I. 5.85–9.27); in the adults group, the mean chest tube removal was 5.56 ± 2.06 days (95% C.I. 4.42–6.70); for the elderly group, it was 10.14 ± 5.58 days (95% C.I. 6.92–13.36). After statistical analysis, we found a statistically significant difference *(p* = *0.038).*

The mean postoperative length of stay (LOS) in the total population was 9.00 ± 5.59 days (95% C.I. 6.87–11.12), greater in the elderly group (12.29 ± 9.70, 95% C.I. 8.27–16.31), and shorter in the adults group (6.44 ± 2.35, 95% C.I. 5.14–7.74). A statistically significant difference was found (*p* = *0.033*) (Table 2).

After over-30-day of follow-up, 26 (89.65%) patients returned to normal activities of daily living, with a mean IADL score equal to 8, without any recurrence of disease and without any significant difference between group. Three (10.35%) patients, all elderly, had a mean IADL score equal to 6.

## Discussion

Our study demonstrates the safety of minimally invasive u-VATS procedure in the treatment of stage II PE.

The postoperative results achieved by the thoracoscopic approach were remarkable, fully satisfying the two main goals of PE treatment:

- a meticulous debridement of the pleural cavity and complete decortication of the visceral and parietal pleura in all 29 patients;
- adequate lung re-expansion in 100% of the series.

Furthermore, we report the first experience on u-VATS approach to stage II PE in elderly population older than 70 years. Our findings demonstrated the same outcomes in terms of efficacy and safety than in adult, showing a longer chest tube stay and hospital stay that we will discuss below.

Until a few years ago, the standard of care for stages II and III PE was represented by thoracotomy, but nowadays MIS gained even more diffusion as reported by several studies which compared thoracotomy and VATS approaches to treat stage II-III PE reporting equivalent results in terms of safety and efficacy [19–22].

Furthermore, VATS achieved the additional benefits of the minimally invasive approach, including reducing postoperative pain and days of chest tube stay with early discharge [19, 20, 22, 23].

Although it seemed to be clear that our approach allowed a safety management of stage II PE, we compared our peri-operative data with the most relevant experiences on m-VATS and open approaches in order to deeply understand benefits and drawbacks of uniportal approach as resumed in Tables 4 and 5.

Comparing our results with studies concerning patients undergoing VATS (either uniportal or multiportal approach) (Tables 4 and 5), it is possible to highlight that our outcomes do not differ from the standard of care, also showing better results in terms of 30-days mortality [24–26].

Concerning preoperative patients’ characteristics, we report a mean age of 67.13 ± 9.96 years, that is 15 years average higher than the average age reported in PE case series [19, 21–33].

Obviously, also comorbidities prevalence, like hypertension, COPD, and cardiovascular diseases, are higher in our population due to older patients [34–36].

Literature data concerning conversion rate to thoracotomy are heterogeneous, ranging from 6% [20, 26, 31] up to 59% [30]. This discrepancy is related either to the progressive learning curve either to the stage of the disease treated.

According to Stefani et al. [31], the probability of thoracotomy raises according to the waiting time for surgery; this finding can be linked to the evolution of stage II to a chronic organized phase (stage III) PE, characterized by multiple loculations, and fibrothorax with diffuse lung entrapment.

Our patients underwent u-VATS 10 days maximum after admission, considering also the low impact of the uniportal approach to patients general conditions with consequent low rate of conversion.

Pleural adhesions, characteristic of a stage III PE, are one of the main limitations to VATS approach in those patients, because they cause a challenging access to the thoracic cavity and the subsequent inability to perform a complete lung decortication [33].

In fact, in our experience, conversion to traditional thoracotomy was only necessary in 1 (6.25%) adult case, due to the toughness of the countless pleural adhesion.

Operating time was confirmed to be in line with the experience of other centers [23, 29, 32, 33].

Regarding postoperative course, we do not report any major complications. As Clavien–Dindo I minor complications, we report an overall complications rate of 62.07%, 40.00% in the adults group and 85.71% in the elderly group in contrast to lower rates reported in other studies [20, 23, 24, 27, 29, 31, 33, 37].

As already declared, our study cohort was older than the cohorts examined by other authors; therefore, the increased rate of minor complications could be related to elderly age and the lack of patient compliance to postoperative respiratory physiotherapy.

In relation to the chest tube removal, we report 7.56 ± 4.50 days (95% C.I. 5.85–9.27), compared to a mean stay of 6.11 ± 2.89 days (95% C.I. 5.92–6.30) in the literature [19, 22–28, 30–32].

Moreover, LOS is comparable with other referral centers experience, as shown in Table 5.

**Table 4:** Literature review—preoperative characteristics

Author, year	Total population	Age, mean ± SD or mean and (range)	Pleural empyema stage	Treatment			
				Chest tube	VATS		Open
					Uniportal	Multiportal	
Landreneau et al. [23]	76	47 [14–78]	II-III	0	0	76	0
Wait et al. [19]	20	VATS 42 ± 20/CT 43 ± 13	II	9	0	11	0
Angelillo Mackinlav et al. (24)	64	VATS 48.9 ± 17.6/ Open 51.1 ± 17.8	II	0	0	31	33
Cassina et al. [27]	45	52 [13–86]	II	0	0	45	0
Roberts et al. [25]	172	53.68 [13–86]	II-III	0	0	66	106
Kim et al. [28]	70	40 ± 15	II-III	0	0	70	0
Lardinois et al. [29]	328	55 [3–92]	II	0	0	178	150
Solaini et al. [30]	110	52 [7–88]	II-III	0	0	110	0
Cardillo et al. [21]	308	VATS 55.8 ± 10.6/Open 57 ± 12.9	II-III	0	0	185	123
Stefani et al. [31]	97	54 [21–83]	II-III	0	0	97	0
Bongiolatti et al. [32]	64	57.8 ± 16.4	II-III	0	30	0	34
Ismail et al. [22]	35	57.26 ± 18.29	I-II-III	0	35	0	0
Semenkovich et al. [33]	4095	CT 64 [11]/ VATS 56 [45–69]/ Open 57 [47–69]	II	1563	1313	0	1219
van Middendorp et al. [26]	186	u-VATS 60 ± 15.2/ m-VATS 59.6 ± 14.8	II-III	0	49	137	0

SD, standard deviation; CT, chest tube; VATS, video-assisted thoracoscopy; m-VATS, multiportal VATS; u-VATS, uniportal VATS.

**Table 5:** Literature review: intra- and postoperative characteristics

Author, year	Total population	Conversion rate (%)	Complications (%)	Operative time (min), mean ± SD or mean and (range)	LOS (days), mean ± SD or mean and (range)	Chest tube stay (days), mean ± SD or mean and (range)	30-day mortality (%)
Landreneau et al. [23]	76	17.1	NA	NA	7.4 ± 7.2	3.3 ± 2.9	6.6
Wait et al. [19]	20	0	VATS 0/CT 11.11	NA	VATS 8.7 ± 0.9/ CT 12.8 ± 1.1	VATS 5.8 ± 1.1/ CT 4.2 ± 1.8	VATS 9.09/CT 11.11
Angelillo Mackinlav et al. [24]	64	9.67	VATS 16.13/Open 15.15	VATS 119 ± 32.5/ Open 123 ± 25.8	VATS 6.7 ± 3.0/ Open 11.6 ± 9.1	VATS 4.2 ± 1.5/ Open 6.1 ± 2.3	VATS 3.22/Open 0
Cassina et al. [27]	45	10	16	NA	10.7 [6–140	7.1 [4–140]	3
Roberts et al. [25]	172	NA	VATS 9/ Open 21	NA	15.3	10.5	1.8
Kim et al. [28]	70	7.14	NA	79.5 ± 15	5.7 ± 6	5 ± 2	0
Lardinois et al. [29]	328	44.38	9	NA	NA	NA	VATS 3/ Open 4
Solaini et al. [30]	110	8.2	10.9	120 [35–220]	7.1 [5–17]	6 [3–25]	0
Cardillo et al. [21]	308	5.94	VATS 18.3/ Open 25.2	VATS 70 ± 7.4/ Open 79.6 ± 6.8	VATS 8.6 ± 1.8/ Open 10 ± 7.8	NA	VATS 0/ Open 3.2
Stefani et al. [31]	97	59	VATS 12.5/ Open 32	VATS 146 [90–210]/ Open 162 [80–255]	VATS 8.3 [3–30]/ Open 8.4 [3–44]	VATS 4.4 [2–12]/ Open 5 [2–40]	NA
Bongiolatti et al. [32]	64	10	VATS 10/ Open 47	VATS 116 ± 28/ Open 135 ± 43	VATS 6.7 ± 1.9/ Open 12.2 ± 4.7	VATS 5.6 ± 1.4/ Open 10.6 ± 4.2	0
Ismail et al. [22]	35	0	22.8	128.29 ± 8.98	15.6 ± 8.98	8.91 ± 7.01	0
Semenkovich et al. [33]	4095	15	CT 15.4/ VATS 4.7/ Open 6	NA	CT 14 [9–22]/ VATS 12 [9–19]/ Open 15 [10–21]	NA	CT 18.3/ VATS 5.4/ Open 6.8
van Middep et al. [26]	186	u-VATS 0/ m-VATS 3	u-VATS 18/ m-VATS 10	u-VATS 65.3 ± 17.9/ m-VATS 56.4 ± 23.3	u-VATS 18.9 ± 12.8/ m-VATS 20.1 ± 14.7	u-VATS 6.4 ± 4.3/ m-VATS 8.9 ± 6.2	u-VATS 8/ m-VATS 6

SD, standard deviation; CT, chest tube; VATS, Video-assisted thoracoscopy; m-VATS, multiportal VATS; u-VATS, uniportal VATS; LOS, length of stay

The effectiveness of u-VATS approach was investigated in the elderly also though the IADL scale. To our knowledge, this is the first report in thoracic surgery where daily living activities after emergency surgery were evaluated, with such successful outcomes.

As declared above, we found a significant longer chest tube stay and hospital stay in the elderly population. This result is due to the high prevalence of comorbidities associated with increased difficulty in the early removal of chest tubes.

Furthermore, the geographical and social limitation of Molise, already discussed in other experience [38], caused a longer hospital stay due to the lack of social supports and peripheral care, with, consequent, higher hospital related complications [39].

## Limitations

We understand that our study shows some limitations. First, the institution of a new thoracic team into a general surgery department does not allow comparative analysis with the previous treatment. Furthermore, the learning curve on u-VATS, acquired by authors during previous experiences, allowed to directly start the u-VATS approach in PE patients.

Moreover, the limited patient sample and the lack of a comparison group (u-VATS *vs.* thoracotomy or fibrinolytic therapy) are the major limitations of this study. However, based on our experience, we are able to confirm the feasibility and safety of the u-VATS procedure in the treatment of PE because it permits an easy performance of a complete debridement and decortication with a very low conversion rate risk.

## Conclusions

Despite the absence of international guidelines recognizing a precise role for u-VATS in the treatment of stage II PE, our experience recommends u-VATS as a safe alternative in patients with fibrinopurulent disease also after failure of conservative treatments. Furthermore, early u-VATS approach may allow a lower risk of progression to stage III PE and septic complications, also in the elderly.

Further comparative multicenter analyses are advocated to set the u-VATS approach as standard of care.

## Supplementary Information

**Additional file 1.** PROTOCOLLO DECISIONALE PER IL TRATTAMENTODELL’ANEMIA POST-OPERATORIA.
