# Supplementary material for: The uniportal VATS in the treatment of stage II pleural empyema: a safe and effective approach for adults and elderly patients—a single-center experience and literature review
Source: World J Emerg Surg. 2022 Aug 29;17:46. doi: 10.1186/s13017-022-00438-8 (PMC9423701; doi:10.1186/s13017-022-00438-8)
Supplement: Supplementary file 1 — Additional file 1. PROTOCOLLO DECISIONALE PER IL TRATTAMENTODELL’ANEMIA POST-OPERATORIA. [file 13017_2022_438_MOESM1_ESM.docx]

**
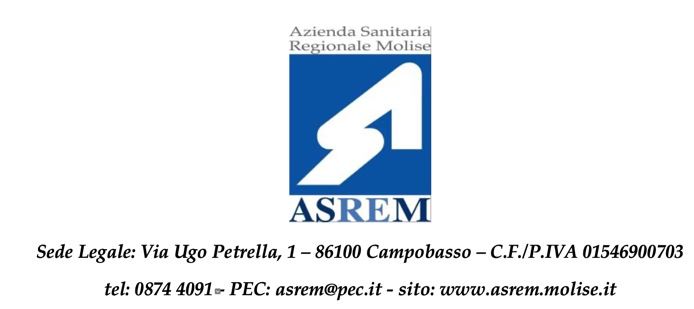
**

**PROTOCOLLO DECISIONALE PER IL TRATTAMENTO**

**DELL’ANEMIA POST-OPERATORIA**

**U.O.C. di Chirurgia Generale – P.O. “A. Cardarelli”**

**Scopo**

Il presente documento vuole essere un ausilio utile al personale medico-infermieristico per utilizzare in modo attento e motivato la risorsa sangue così anche da ottenerne il miglior rapporto costo- efficacia.
Attualmente non esistono studi controllati, prospettici, randomizzati che dimostrino in modo conclusivo l’utilità degli emocomponenti.

**Applicazione**

Il protocollo si applica ogni qual volta si ritenga necessario ricorrere alla somministrazione di **emocomponenti omologhi** nei **pazienti adulti** chirurgici, in elezione o urgenza.

**Destinatari**

Tale protocollo è indirizzato a medici ed infermieri dell’U.O.C di Chirurgia Generale del P.O. “A. Cardarelli” coinvolti nell’ impiego clinico degli emocomponenti.

**Contenuto**

1. **Raccomandazione per la trasfusione di concentrati eritrocitari**
   1. *Terapia trasfusionale dell’anemia post-operatoria*
   2. *Maximum Surgical Blood Ordering Schedule (MSBOS)*
   3. *Terapia trasfusionale nell’anemia acuta*
2. **Raccomandazione per la trasfusione di concentrati eritrocitari**

La trasfusione di concentrati eritrocitari (CE) è indicata per aumentare rapidamente l’apporto di ossigeno ai tessuti, quando la concentrazione di emoglobina è bassa e/o la capacità ossiforetica è ridotta, in presenza di meccanismi di compenso fisiologici inadeguati.

Non esiste un valore assoluto di Hb che imponga la trasfusione; ogni paziente va valutato in base alla sua capacità di tollerare l’anemia: i pazienti cardiopatici e broncopneumopatici hanno rispettivamente una maggiore richiesta ed una minore saturazione di O2 e pertanto hanno una maggiore necessità di ripristinare il potere ossiforetico del sangue.

- 1. *Terapia trasfusionale dell’anemia post-operatoria*

I criteri di compatibilità gruppo-ematica per la trasfusione di globuli rossi sono riportati in tabella 1.
Non ci sono parametri vincolanti per guidare la necessità di trasfondere globuli rossi. La decisione di trasfondere è complessa e dipende da diversi fattori:

• causa dell’anemia

• dalla severità e dalla cronicità dell’anemia

•dalla capacità del paziente di compensare l’anemia

•dall’aspettativa di ulteriori perdite

•dalla necessità di procurare una riserva prima della comparsa della ipossia tessutale.

I rischi della trasfusione devono essere anche bilanciati nei riguardi dei benefici attesi [1].

| **Tabella 1**. Criteri di compatibilità gruppo-ematica | | | | | | | | | |
| --- | --- | --- | --- | --- | --- | --- | --- | --- | --- |
|  | | **DONATORE** | | | | | | | |
|  |  | 0 Rh - | 0 Rh + | A Rh - | A Rh + | B Rh - | B Rh + | AB Rh - | AB Rh + |
| **RICEVENTE** | 0 Rh - | X |  |  |  |  |  |  |  |
|  | 0 Rh + | X | X |  |  |  |  |  |  |
|  | A Rh - | X |  | X |  |  |  |  |  |
|  | A Rh + | X | X | X | X |  |  |  |  |
|  | B Rh - | X |  |  |  | X |  |  |  |
|  | B Rh + | X | X |  |  | X | X |  |  |
|  | AB Rh - | X |  | X |  | X |  | X |  |
|  | AB Rh + | X | X | X | X | X | X | X | X |

In passato il livello di Hb che era preso come indicazione alla trasfusione nel paziente chirurgico era pari a 10 g/dL.
Più recentemente si è passati dalla semplice valutazione di un singolo parametro ad un più complesso criterio valutativo.
Dall’analisi comparativa delle linee guida si evince, inoltre, una generale tendenza ad adottare dei **criteri sempre più restrittivi** nel ricorso alla trasfusione di CE.
*Hebert et al.* in uno studio condotto su pazienti cardiopatici ricoverati in terapia intensiva dimostrano come il mantenimento di un range emoglobinico fra 7 g/dL e 9 g/dL invece che fra 10 g/dL e 12 g/dL non alterava l’outcome e la mortalità, ad eccezione dei pazienti con un infarto miocardico acuto o una angina instabile [2]. Un risultato analogo si è ottenuto in un trial multicentrico che ha coinvolto una popolazione estremamente eterogenea di pazienti ricoverati in terapia intensiva [3]. Inoltre, come descritto in uno studio condotto da *Carson et al*. su 8787 pazienti sottoposti ad intervento chirurgico per frattura di femore, la trasfusione pre-operatoria, in pazienti con valori di Hb > a 8 g/dL, non sembra influenzare la mortalità a 30 giorni dall’intervento [4].
Da tutto ciò si evince come con valori di **Hb > 10 g/dL la trasfusione non è quasi mai appropriata**.
Con un range emoglobinico compreso **fra 6 g/dL e 10 g/dL** la decisione di trasfondere un paziente deve essere supportata dalla concomitante presenza di una clinica positiva per anemia. In pazienti con comorbidità di importante entità, si può attuare una terapia trasfusionale anche in assenza di segni e sintomi di anemia, ma esclusivamente per prevenire eventi avversi gravi. Con **Hb < 6-7 g/dL** la trasfusione è spesso appropriata. In pazienti sani, senza sintomi e segni riferibili all’anemia, la soglia trasfusionale può essere ulteriormente abbassata [5-8].
Quindi, la decisione di trasfondere CE dipende da:

• concentrazione di Hb (tabella 2);

• tipo di intervento;

• entità e rapidità delle perdite ematiche;

• condizioni cliniche del paziente (età, malattie cardiache, respiratorie);

| **Tabella 2.** Criteri decisionali per la trasfusione perioperatoria | |
| --- | --- |
| **Livello di emoglobina** | **Indicazione** |
| >10 g/dL | CE difficilmente indicati |
| 7-10 g/dL | Procedere alla valutazione di: |
|  | • durata dell’anemia  • intervento chirurgico  • perdite ematiche previste  • comorbidità |
| <7 g/dL | CE indicati in relazione alla clinica |

**1.2** *Maximum Surgical Blood Ordering Schedule (MSBOS)*

La letteratura più recente [6] abbassa il trigger trasfusionale a 6 g/dL nel paziente giovane, asintomatico specialmente quando l’anemia è acuta.
In tutti i casi nei quali è prevedibile e/o necessario il ricorso all’uso di sangue allogenico, si suggerisce di richiedere al Servizio Trasfusionale un numero di unità di CE non eccedente l’indicazione **MSBOS** per quel determinato intervento.

Si prendono come riferimento le indicazioni del *British Committee for Standards in Haematology Blood Transfusion Task Force [9],* che riporta la richiesta massima accettabile in condizioni operatorie standard e in presenza di una buona pratica trasfusionale (tabella 3).

| **Tabella 3**. Proposta di Richiesta massima di sangue per tipo di intervento - MSBOS | |
| --- | --- |
| **Chirurgia Generale** | **N° massimo di unità** |
| Colecistectomia | T&S |
| Laparotomia esplorativa | 2 |
| Addominoplastica | T&S |
| Gastrostomia ed enterostomia | T&S |
| Resezioni gastriche | T&S |
| Gastrectomia totale | 2 |
| Splenectomia | T&S |
| Resezione del retto per via addomino-perineale | 4 |
| Resezione anteriore del retto | 2 |
| Resezioni ileali | 4 |
| Resezioni coliche, emicolectomia, colectomia | 2 |
| Mastectomia semplice | T&S |
| Tiroidectomia | T&S |
| **Chirurgia Toracica** | **N° massimo di unità** |
| Biopsia polmonare | T&S |
| Pneumectomia | 2 |
| Lobectomia | 2 |
| Decorticazione pleurica | 2 |
| Toracotomia esplorativa | T&S |

Tabella 3. T&S, Type and Screen, tipizzazione con determinazione del gruppo AB0 e del tipo Rh.

***1.3*** *Terapia trasfusione nell’anemia acuta*

L’ipovolemia, evento associabile all’anemia, deve essere sempre corretta con **soluzioni di cristalloidi o di colloidi** prima di procedere con le trasfusioni.
La **concentrazione di Hb** rappresenta solo una delle variabili da considerare. Va ricordato che i pazienti con anemia acuta possono presentare valori di Hct normali, o addirittura elevati, finché il volume plasmatico non viene ripristinato. Diviene quindi molto importante la **valutazione clinica** del paziente.
L’**entità̀ della perdita ematica** è l’altro importante parametro da considerare (tabella 4).
Ad eccezione delle situazioni di emergenza occorre rivalutare il paziente dopo ogni singola trasfusione al fine di minimizzare l’impiego di emazie da donatore.

| **Tabella 4**. Reazione all’emorragia acuta di crescente gravità e indicazioni trasfusionali | | | |
| --- | --- | --- | --- |
| **Classe di emorragia** | **Riduzione del vol. ematico tot.** | **mL*** | **Provvedimenti terapeutici** |
| Classe I | <15% | <750 | • Soluzioni cristalloidi/colloidi  • CE necessari SOLO se preesistente anemia |
| Classe II | 15-30% | 750-1500 | • Soluzioni cristalloidi/colloidi  • CE necessari SOLO se preesistente anemia e/o malattia cardiopolmonare |
| Classe III | 30-40% | 1500-2000 | • Soluzioni cristalloidi/colloidi  • CE probabilmente necessari |
| Classe IV | >40% | >2000 | • Soluzioni cristalloidi/colloidi in infusione rapida  • CE necessari, in infusione rapida |

Tabella 4. mL* Calcolati su un individuo adulto di 70 Kg e con un volume ematico totale di 5000 mL

References

1. Murphy, M.F., et al., *Guidelines for the clinical use of red cell transfusions.* Br J Haematol, 2001. **113**(1): p. 24-31.

2. Hébert, P.C., et al., *Is a low transfusion threshold safe in critically ill patients with cardiovascular diseases?* Crit Care Med, 2001. **29**(2): p. 227-34.

3. Hébert, P.C., et al., *A multicenter, randomized, controlled clinical trial of transfusion requirements in critical care. Transfusion Requirements in Critical Care Investigators, Canadian Critical Care Trials Group.* N Engl J Med, 1999. **340**(6): p. 409-17.

4. Carson, J.L., et al., *Perioperative blood transfusion and postoperative mortality.* Jama, 1998. **279**(3): p. 199-205.

5. *Practice Guidelines for blood component therapy: A report by the American Society of Anesthesiologists Task Force on Blood Component Therapy.* Anesthesiology, 1996. **84**(3): p. 732-47.

6. *Practice guidelines for perioperative blood transfusion and adjuvant therapies: an updated report by the American Society of Anesthesiologists Task Force on Perioperative Blood Transfusion and Adjuvant Therapies.* Anesthesiology, 2006. **105**(1): p. 198-208.

7. Hill, S.R., et al., *Transfusion thresholds and other strategies for guiding allogeneic red blood cell transfusion.* Cochrane Database Syst Rev, 2002(2): p. Cd002042.

8. Spahn, D.R., *Perioperative Transfusion Triggers for Red Blood Cells.* Vox Sang, 2000. **78 Suppl 2**: p. 163-6.

9. *Guidelines for implementation of a maximum surgical blood order schedule. The British Committee for Standards in Haematology Blood Transfusion Task Force.* Clin Lab Haematol, 1990. **12**(3): p. 321-7.
